# A posteriori accuracy estimation of ultrasonic vector-flow mapping (VFM)

**DOI:** 10.1007/s12650-016-0413-3

**Published:** 2017-02-17

**Authors:** Tomohiko Tanaka, Rei Asami, Ken-ichi Kawabata, Kunio Hashiba, Takashi Okada, Tomohide Nishiyama

**Affiliations:** 1 Research and Development Group, Hitachi, Ltd., 1-280 Higashi-Koigakubo, Kokubunji, Tokyo Japan; 2 Healthcare Business Unit, Hitachi, Ltd., 3-1-1 Higashi-Koigakubo, Kokubunji, Tokyo Japan

**Keywords:** Doppler ultrasound, Ultrasonics, Flow imaging, Cardio-hemodynamics, Vector-flow mapping

## Abstract

**Abstract:**

A novel method, called a posteriori “VFM accuracy estimation” (VAE), for resolving an intrinsic VFM problem is proposed. The problem is that VFM uncertainty can easily vary according to blood flows through an echocardiographic imaged plane (i.e., “through-plane” flows), and it is unknown. Knowing the VFM uncertainty for each patient will make it possible to refine the quality of VFM-based diagnosis. In the present study, VAE was derived on the basis of an error-propagation analysis and a statistical analysis. The accuracy of VAE with a pulsatile left-ventricle phantom was experimentally investigated for realistic cases with through-plane flows. VAE was validated by comparing VFM uncertainty (S.D.) estimated by VAE with VFM uncertainty measured by particle-image velocimetry (PIV) for different imaged planes. VAE accurately estimated the S.D. of VFM uncertainty measured by PIV for all cases with different image planes (*R* > 0.6 and *p* < 0.001). These findings on VFM accuracy will provide the basis for widespread clinical application of VFM-based diagnosis.

**Graphical Abstract:**

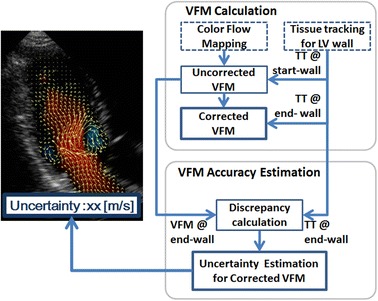

## Introduction

**Fig. 1:**
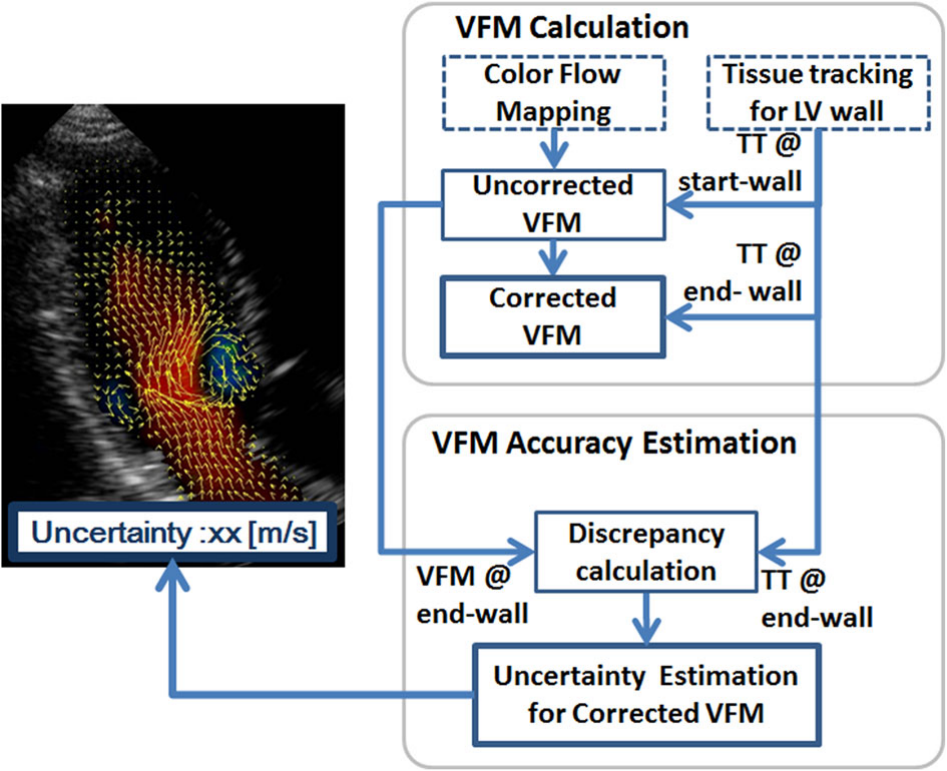
Schematics of a posteriori method for evaluating VFM accuracy. The *left figure* shows a typical ultrasound image for a transmitral flow from the left atrium to the left ventricle. The *background blue/red color* denotes the direction of the flows measured by the color-Doppler technique. The *yellow vectors* are obtained by VFM. Also, VAE method focuses on estimating the uncertainty of the obtained vectors as in a box showing “uncertainty xx (m/s)”. The *right figure* is a flow chart of VFM derivation

Vector-flow mapping (VFM) utilizing ultrasound, as shown in Fig. 1 (left), is expected to open up new routes for very early diagnosis of cardiac dysfunctions, because flows in the left ventricle (LV) are immediately affected by changes in cardiac performance (Sengupta et al. 2012; Munoz et al. 2013). Recently, intracardiac flow patterns, which provide clinical information concerning the functional status of the heart, have been investigated using cardiac magnetic resonance (CMR) (Kim et al. 1995; Kilner et al. 2000; Markl et al. 2005; Gharib et al. 2006; Markl et al. 2011; Gupta et al. 2012), echocardiographic particle-image velocimetry (echo-PIV) (Sengupta et al. 2007, 2006; Hong et al. 2008), and VFM (Uejima et al. 2010; Garcia et al. 2010; Itatani et al. 2013). Compared with CMR and echo-PIV, VFM quickly and non-invasively obtains 2D flow vectors in the LV. VFM estimates cross-beam velocities using color-Doppler velocities on the basis of mass-conservation of fluids under a nontrivial assumption, i.e., planar flow (Uejima et al. 2010; Garcia et al. 2010; Itatani et al. 2013; Ohtsuki and Tanaka 2006), although flows in the LV are three-dimensional (Kilner et al. 2000). Violation of this assumption due to through-plane flows, which depend on imaging conditions such as LV shapes, imaging views, and pulsatile phases, would degrade VFM accuracy.

VFM has been experimentally validated by Garcia et al. (2010) and also by our group (Tanaka et al. 2012; Asami et al. 2016). Garcia et al. reported that the error due to the planar-flow assumption is about 15%, and Asami et al. showed that the VFM uncertainty (S.D.) is less than 10% of the color range under their validation conditions. However, the results of these VFM validations are valid only for the validation cases, because the accuracy, or uncertainty, of VFM may easily change due to the through-plane flows. Thus, the validity of VFM obtained in clinical situations is unfortunately unknown. To make VFM clinically practical, knowing the validity of VFM is essential.

To resolve this problem, the objective of the present study is to establish a posteriori method for estimating the accuracy of each VFM measurement, hereafter, “VFM accuracy estimation” (VAE) (shown in the right side of Fig. 1). Knowing the uncertainty in each VFM measurement makes it possible to refine the quality of VFM-based diagnosis and reject or retake VFM measurements with large uncertainties. VAE was analytically derived by error-propagation and statistical analyses, and experimentally validated using an LV phantom.

## Methods

### Review of VFM derivation using continuity equation

**Fig. 2:**
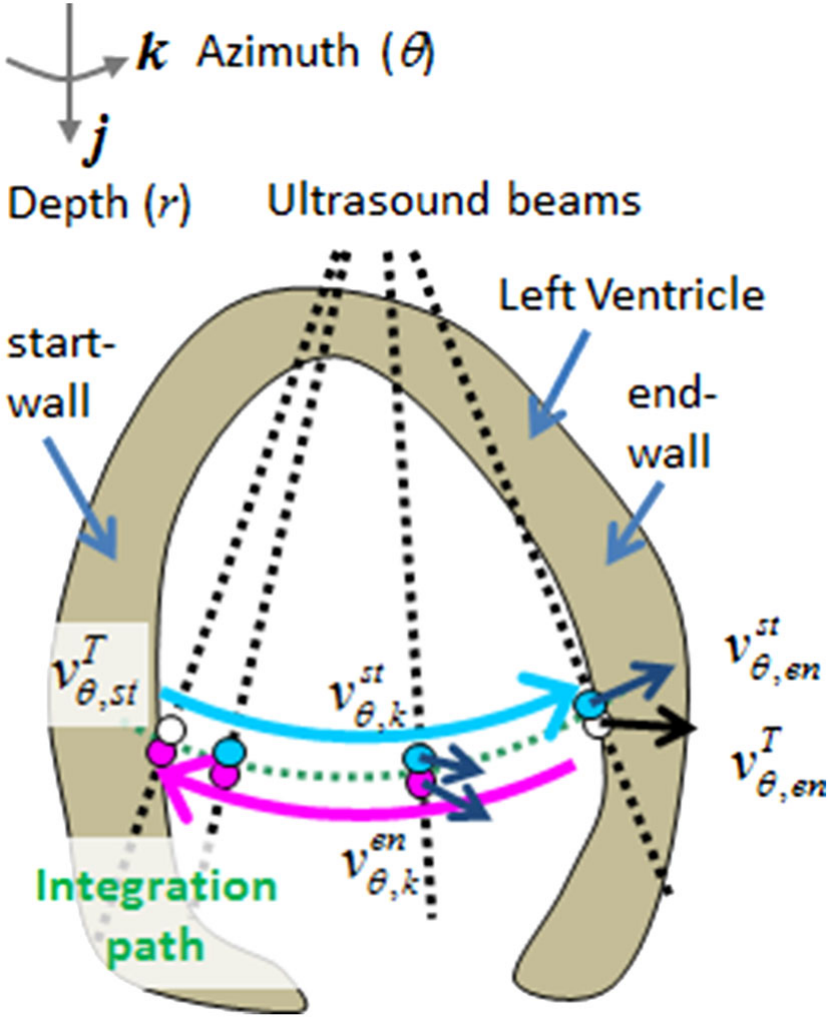
Schematic of VFM calculation for an integration path. Cross-beam, or azimuthal, velocities are calculated by successively integrating the mass-conservation equation. The wall used for the BC is called the “start wall.” The uncorrected VFM velocities starting from the start wall are displayed as *cyan arrows*. Mainly due to errors in the VFM calculation, resultant VFM azimuth velocity at the other side of the wall (end wall), $v_{{\theta ,{\text{en}}}}^{\text{st}} (r)$, is not the same as the wall azimuthal velocity measured by tissue tracking, $v_{{\theta ,{\text{en}}}}^{T} (r)$, because of the error accumulations during the integration procedures. Therefore, another uncorrected VFM is calculated from the end wall as displayed as *magenta arrows*

Before VAE is introduced, the VFM calculations (shown in the upper half of the flow chart in Fig. 1) are briefly reviewed (Itatani et al. 2013; Asami et al. 2016). First, an uncorrected VFM is derived. Cross-beam (or azimuthal) velocities are calculated by successively integrating the mass-conservation equation in the azimuthal direction on the basis of color flow mapping (CFM), where the boundary-wall velocity is measured by tissue tracking (TT), as shown in Fig. 2. Since the scanning manner of an ultrasound sector probe fits the cylindrical coordinate system, the mass-conservation equation in the cylindrical coordinate system is expressed as

1 $$r\partial_{r} v_{r} + v_{r} + \partial_{\theta } v_{\theta } + r\partial_{z} v_{z} = 0,$$

where *v* represents flow velocity, subscript *r* denotes radial direction (or beam direction), *θ* represents azimuthal direction (or cross-beam direction), and *z* denotes through-plane direction. Equation (1) is simplified and integrable with respect to $v_{\theta }$ by assuming that the through-plane derivative term is negligible compared with the other terms. This assumption is hereafter called “planer-flow assumption.” The azimuthal velocity is written as

2 $$v_{\theta } (r,\theta ) = v_{{\theta ,{\text{st}}}} (r) + \int_{{\theta_{st} }}^{\theta } {\partial_{\theta } v_{\theta } } {\text{d}}\theta ,$$

where the inside of the integral in Eq. (2) consists of quantities in the radial direction and can be obtained using the conventional CFM method as

3 $$\partial_{\theta } v_{\theta } = - r\partial_{r} v_{r} - v_{r} .$$

Subscript, st (or en), in Fig. 2 and Eq. (2) denotes the position of the boundary conditions (BCs) for the VFM integration, namely, heart-wall velocities at wall surfaces measured by tissue tracking. The wall used for the BC is called the “start (or end) wall.” Uncorrected VFM velocity starting from the start wall, $v_{\theta }^{\text{st}} (r,\theta )$, is given as

4 $$v_{\theta }^{\text{st}} (r,\theta ) = v_{{\theta ,{\text{st}}}}^{T} (r) + \int_{{\theta_{st} }}^{\theta } {\partial_{\theta } v_{\theta } } {\text{d}}\theta .$$

Note that fluid velocity at the LV wall by VFM is the same as the wall-surface velocity measured by tissue tracking under the non-slip condition. However, mainly due to errors in the VFM calculation, resultant VFM azimuthal velocity at the other side of the wall (end wall), $v_{{\theta ,{\text{en}}}}^{\text{st}} (r)$, differs from wall azimuthal velocity measured by tissue tracking, $v_{{\theta ,{\text{en}}}}^{T} (r)$, because of error accumulations during the integration procedures. Velocity discrepancy *A*(*r*) is defined as

5 $$A(r) = v_{{\theta ,{\text{en}}}}^{\text{st}} (r) - v_{{\theta ,{\text{en}}}}^{T} (r).$$

Another uncorrected VFM velocity integrated from the end wall, $v_{\theta }^{\text{en}} (r)$, described in Eq. (6), also differs from $v_{\theta }^{\text{st}} (r,\theta )$, although both velocities should ideally be the same; namely,

6 $$v_{\theta }^{\text{en}} (r,\theta ) = v_{{\theta ,{\text{en}}}}^{T} (r) - \int_{\theta }^{{\theta_{en} }} {\partial_{\theta } v_{\theta } } {\text{d}}\theta .$$

Next, the derivation of the corrected VFM velocity is reviewed using the above-described uncorrected VFMs. Garcia et al. (Garcia et al. 2010) proposed a method for reducing the error accumulations by combining the two velocity fields with a weighted function, *W*, as described in Eq. (7). The corrected velocity field is given as

7 $$v_{\theta }^{C} (r,\theta ) = (1 - W)v_{\theta }^{\text{st}} (r,\theta ) + Wv_{\theta }^{\text{en}} (r,\theta ).$$

In the present study, the following simple linearly weighted function (Itatani et al. 2013) is used as *W*:

8 $$W = \frac{{\theta - \theta_{\text{st}} }}{{\theta_{\text{en}} - \theta_{\text{st}} }}.$$

Note that the corrected velocity in the discretized form, $v_{\theta }^{C} (r,\theta )$, can be rewritten using *A*(*r*) (Eq. (5)) as

9 $$v_{\theta }^{C} (r) = v_{\theta ,k}^{\text{st}} (r) - \frac{k}{N}A(r).$$

Subscript, *k,* denotes the *k*th point in a discretized integration path, as described in Fig. 2, and *N* is the total number of integration points.

### Proposed method

#### Underlying idea for VAE

The goal of the proposed method is to estimate VFM accuracy as accuracy information. The general description of uncertainty of the corrected VFM velocity, $v_{\theta }^{C}$, with confidence intervals (CI) is given as

10 $$\varepsilon = \mu_{{\Delta v_{\theta }^{C} }} \pm t\sigma_{{\Delta v_{\theta }^{C} }} ,$$

where $\varepsilon$ is uncertainty, and *t* is a student value. Uncertainty can be with 95% CI by taking *t* of 1.96. Expressions $\mu_{\left( \cdot \right)}$ and $\sigma_{\left( \cdot \right)}$ denote the mean and standard deviation for an arbitral parameter, $\left( \cdot \right)$, such as corrected VFM vector error, $\Delta v_{\theta }^{C}$, which is an error from the true velocity value, $v_{\theta }^{{}}$, defined as

11 $$\Delta v_{\theta }^{C} = v_{\theta }^{C} - v_{\theta } .$$

Specifically, to estimate $\varepsilon$ from Eq. (10), it is necessary to find the unknowns ($\mu_{{\Delta v_{\theta }^{C} }}$ and $\sigma_{{\Delta v_{\theta }^{C} }}$) with known properties.

#### Derived form

The derived form of the VFM uncertainty (whose detailed formulation is given in Appendix A) is given as

12 $$\varepsilon = \pm t\frac{{\sigma_{A} }}{\sqrt 6 },$$

where $\sigma_{A}$ is standard deviation of velocity discrepancy, *A*. In comparing Eq. (12) with Eq. (10), each term can be written as

13 $$\mu_{{\Delta v_{\theta }^{C} }} \cong 0,$$

14 $$\sigma_{{\Delta v_{\theta }^{C} }} \cong \frac{{\sigma_{A} }}{\sqrt 6 }.$$

The procedure of a posteriori VFM accuracy estimation (VAE) is summarized in the lower half of the flow chart in Fig. 1. From measured *A*, VFM accuracy can be estimated and then fed back to sonographers. Note that the uncertainty for the corrected VFM was calculated based on the discrepancy, *A*, calculated from the uncorrected VFM fields as defined in Eq. (5).

### Experimental setup

#### Overview of experimental setup

**Fig. 3:**
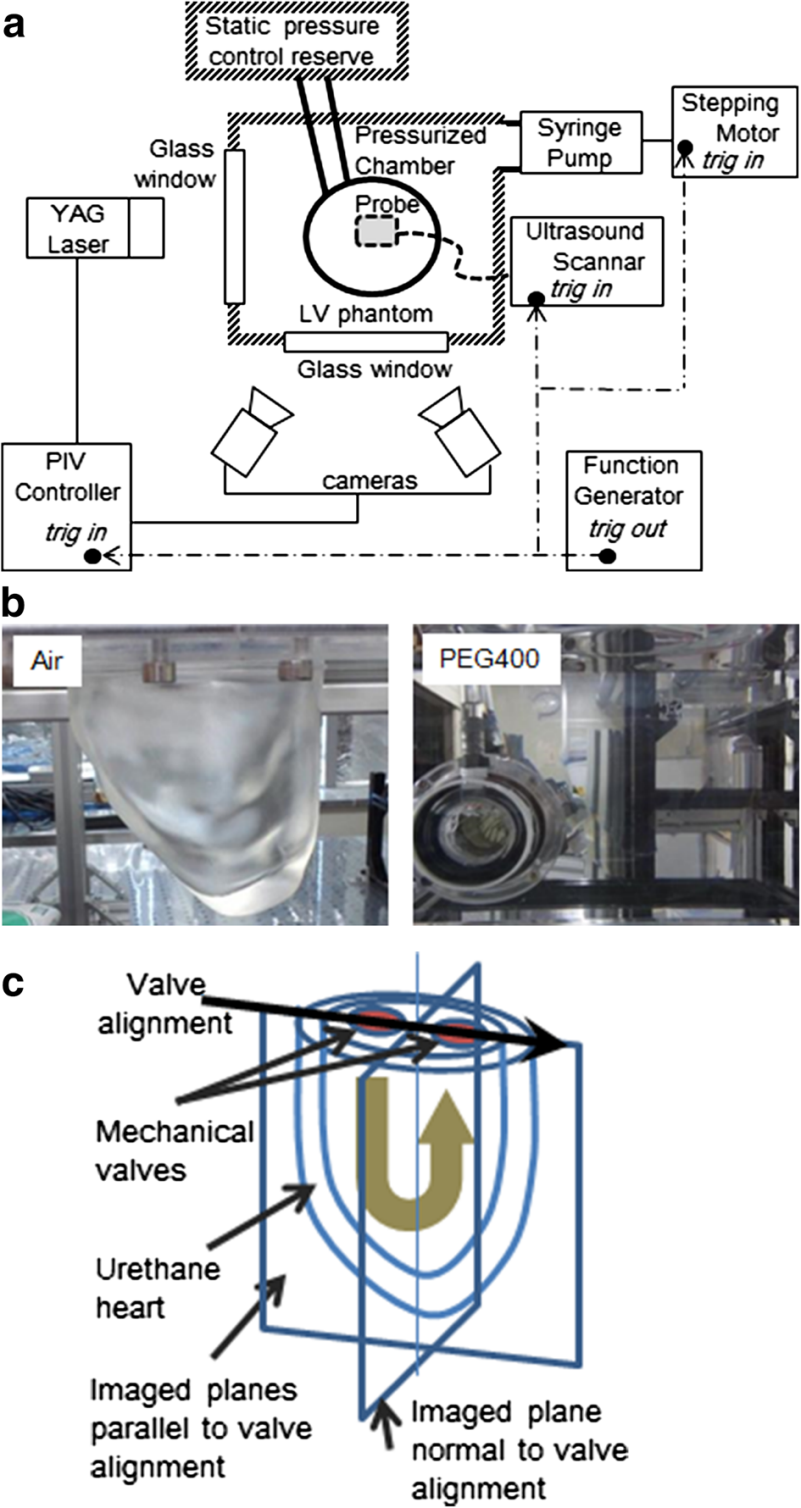
Experimental system: **a** schematics of the experimental setup, which consists of an LV phantom, an ultrasound scanner, and a PIV. A pulse signal mimicking an R-wave generated by an activator synchronizes all three systems at 1 Hz. **b** LV phantom in air (*left*) and in PEG400 (*right*). **c** Schematics of the imaged planes

A top view of the experimental facility, which consists of an LV phantom, an ultrasound scanner, and a PIV, are shown in Fig. 3. A complete description of the facility can be found in our article (Asami et al. 2016). A pulse signal mimicking an R-wave in the electrocardiogram, which was generated using an activator (33220A, Agilent Technologies, Inc., USA), activated all three systems at 1 Hz.

#### Particle-image velocimetry

The PIV acquired 2D velocity fields in the phantom. A Raypower 5000 PIV Nd:YAG laser (continuous 5 W at 532 nm) illuminated tracers (Expancel^®^ 80, Japan Fillite Co., Ltd., Japan) in the test section and DANTEC Dynamics SpeedSense1010 cameras with 50-mm micro-Nikkor lenses captured the tracer images at a frame rate of 250 Hz. To cancel out a background noise, only tracer images were extracted (by subtracting the background images). PIV vectors were calculated using commercial software (Dynamic Studio, Dantec Dynamics, A/S, Denmark). A standard cross-correlation algorithm with three-point Gaussian fitting (Willert and Gharib 1991) was used for subpixel accuracy. The vector spacing was set to about 0.4 × 0.7 mm using 8 × 32-pixel interrogation windows with 50 and 75% overlaps. To evaluate the degradation of VFM accuracy, the VFM measurements were compared with those obtained by the PIV, which provides accurate 2D velocity components in a plane. The uncertainty of the three-point Gaussian fitting is typically expected to be about 0.2 pixels.

#### Left-ventricular phantom

The LV phantom was made of polyurethane resin on the basis of 3D LV shape data (model No. 2, Virtual Anatomia, SGI Japan, Ltd., Japan). It was 1.6-times larger than the original LV data. Mechanical valves were attached as the aortic and mitral valves. An optically transparent phantom was created for the PIV measurements. Since the refraction index of the phantom is about 1.47, the fluid used was PEG 400, which has almost the same refraction index as that of the phantom. Both the inside and outside of the phantom were filled with PEG 400 so that the phantom would be optically transparent (Fig. 3b). The phantom was passively pulsated by changing its chamber pressure, which was controlled by a pressure piston (F14-10, Yamaha Motor Co., Ltd., Japan) with stroke volume of about 75 cc. The chamber on the lower side had an acoustic window with an ultrasound probe attached to the window. The validity of VAE was examined in the two different image planes, one parallel and one normal to the valve alignment, as shown in Fig. 3c.

#### Ultrasound scanner

An ultrasound scanner (ProSound^*®*^ *α*10, Hitachi, Ltd., Japan) with a sector probe (UST-52105, Hitachi, Ltd., Japan) acquired color Doppler and B-mode images of about 15 heartbeats for each experimental case. To compare the VFM and PIV vectors, the same calibration board was used to unify the coordinate system. Both spatial and temporal resolutions matched. Since the obtained VFM spatial resolution was higher than the PIV resolution, the VFM vectors were spatially averaged in accordance with the PIV grid size. On the other hand, eight frames of the PIV results at the same phase were averaged to increase the accuracy of the PIV vectors.

The VFM error was measured as a S.D. of the difference between VFM and PIV azimuth velocities in the entire phantom domain. The measured VFM error was compared with the VFM error estimated by VAE given in Eq. (14). S.D. generally expresses the width of a probability density function (PDF) of a variable, and corresponding PDFs were calculated for 15 beats data sets. In VAE, the S.D. of VFM uncertainty, or the corrected VFM error, $\Delta v_{\theta }^{C}$, corresponds to the S.D. of *A* divided by $\sqrt 6$. The number of frames, *f*_*n*_, used for each VAE analysis was changed from one to four. The regions defined in Eq. (45) were used for VAE calculation.

Due to the difference in the speeds of sound in tissue and PEG400, the resultant VFM velocities calculated by the ultrasound scanner, $\overrightarrow {{v^{\text{eqp}} }}$, were modified by simply multiplying the resulted vectors by correction factor $C_{\text{f}}$ as follows:

15 $$\overrightarrow {v} = C_{\text{f}} \overrightarrow {{v^{\text{eqp}} }} ,$$

16 $$C_{\text{f}} = \frac{{c_{\text{P}} }}{{c_{\text{b}} }},$$

where $c_{\text{b}}$ and $c_{\text{P}}$ are the speeds of sound for bodies, 1530 m/s, and for PEG 400, 1610 m/s. The justification for the correction of the speed of sound is briefly described in Appendix B.

## Results

### Comparison of VFM and PIV

**Fig. 4:**
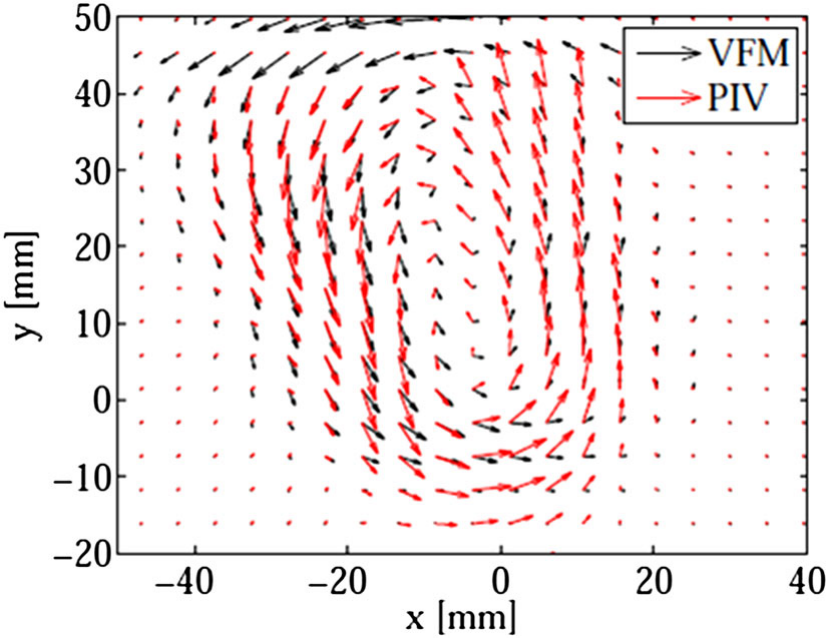
Comparison between VFM (*black*) and PIV (*red*) vectors for the case in which the image plane is parallel to the valve alignment. The apex is at the bottom

A vector map for the case in which the image plane was parallel to the valve alignment is shown in Fig. 4. Overall, the VFM vectors agree well with the PIV vectors. Further validation results for the VFM can be found in other articles (Tanaka et al. 2012; Asami et al. 2016).

### Probability density function

**Fig. 5:**
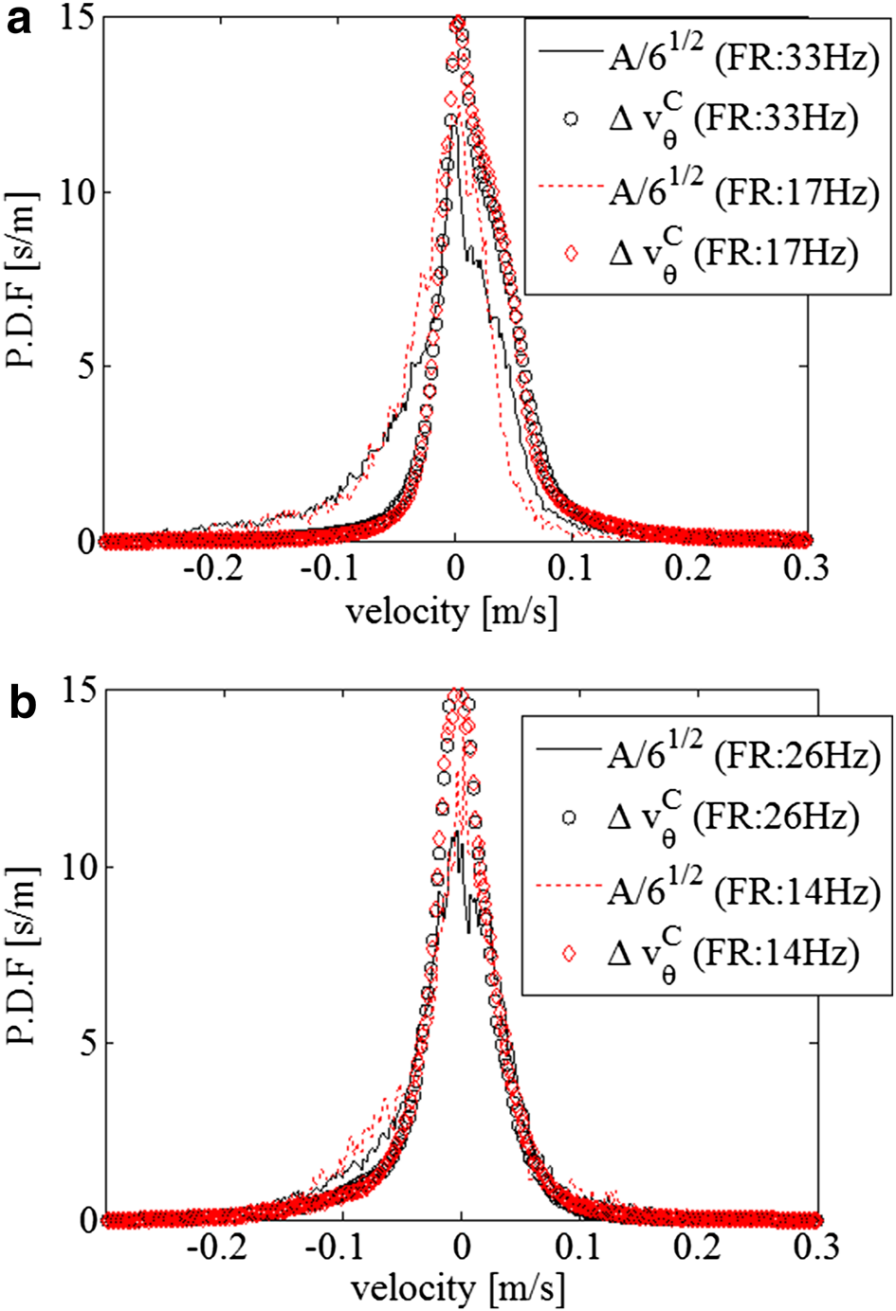
Probability density function of the corrected VFM uncertainty for imaged planes **a** parallel and **b** normal to the valve alignment

Probability density functions (PDFs) of the corrected VFM error, $\Delta v_{\theta }^{C}$, in the imaged planes (a) parallel and (b) normal to the valve alignment with different VFM frame rates are shown in Fig. 5. The corrected VFM errors were determined by comparing velocities obtained by VFM with those obtained by PIV. To obtain the PDFs, a total of 15 heartbeat datasets for each condition were analyzed. For comparison, distributions of quantity, $A/\sqrt 6$, were also plotted on the basis of Eq. (14).

### Correlation between measured and estimated uncertainties

**Fig. 6:**
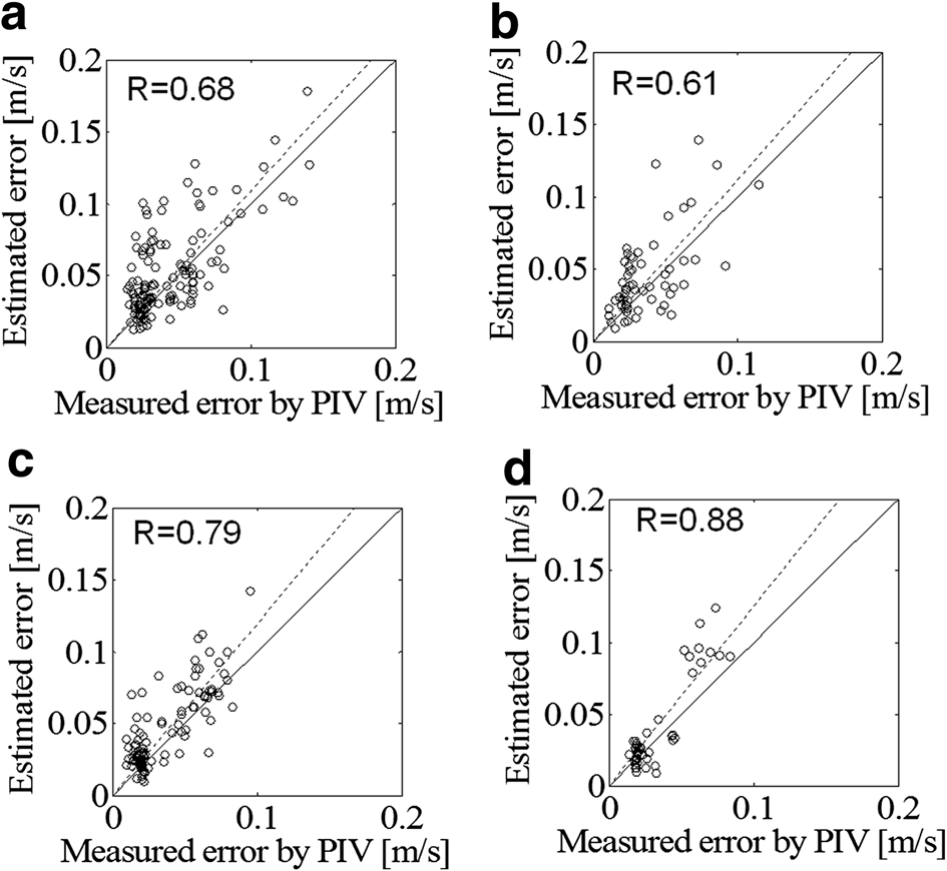
Correlation of measured and estimated uncertainties for cases with imaged plane **a** parallel to the valve alignment at a frame rate of 33 Hz, **b** parallel to the valve alignment at a frame rate of 17 Hz, **c** normal to the valve alignment at a frame rate of 26 Hz, and **d** normal to the valve alignment at a frame rate of 14 Hz. The *solid line* depicts a line with slope of unity. The *dashed line* denotes the fitted line of the correlation plots

**Fig. 7:**
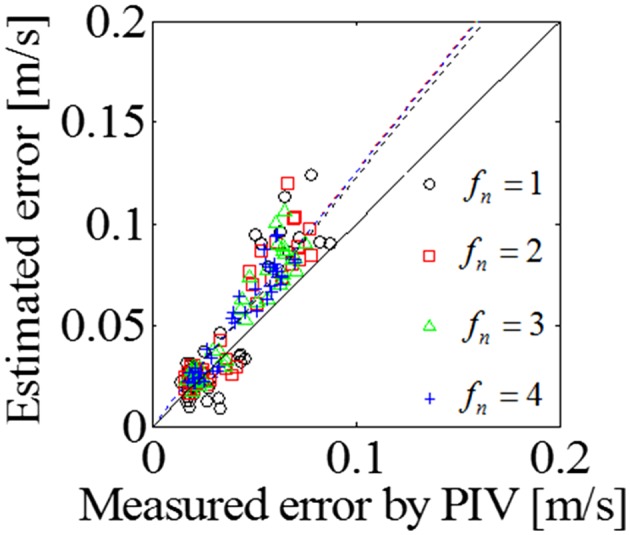
Correlation of measured and estimated uncertainties for cases with imaged plane normal to the valve alignment at a frame rate of 14 Hz using several (from 1 to 4) frames. The *solid line* depicts a line with slope of unity. The *dashed line* denotes the fitted line of the correlation plots

To quantitatively validate VAE, calculated correlations between measured uncertainty (S.D. of $\Delta v_{\theta }^{C}$) and estimated uncertainty (S.D. of ${A \mathord{\left/ {\vphantom {A {\sqrt 6 }}} \right. \kern-0pt} {\sqrt 6 }}$) are plotted in Fig. 6. The solid line depicts a line with slope of unity. The dashed line denotes the fitted line of the correlation plots. The correlation coefficient of the estimated and measured uncertainties, *R*, is stated in the figure. Overall, the estimated uncertainties agree well with *R* of more than 0.6 for all cases (*p* < 0.001). Figure 7 shows the effects of the number of frames using several (from 1 to 4) frames for case as Fig. 6d. As the number of frames increases, the correlation values increase from 0.88 to 0.93. As more frames were used, the correlation plots converged to the fitted lines.

## Discussion

The PDF shapes of the corrected VFM error, $\Delta v_{\theta }^{C}$, for higher and lower frame rates are almost the same as those plotted in Fig. 6. Imaging with a higher frame rate gives higher temporal, but lower spatial resolution. Since the spatial and temporal resolutions have a trade-off relationship, the frame rate itself may slightly affect the statistical uncertainty, as long as the flows are along the imaged plane are the same. Moreover, velocity discrepancy has the same trend as VFM error regardless of frame rate. For the regions that the errors are small in Fig. 6, the errors estimated by the VAE are relatively large. The velocities are also small in the region where the errors are small. The ultrasound velocity measurements for the low velocities range can be degraded due to a clutter filter (Clutter et al. 2002) used in the ultrasound Doppler measurements, because the clutter filter removes not only signals of slow heart tissues but also signals of slow flow motions.

From Fig. 5, the assumption of Gaussian-error distribution used in VAE (Appendix A) is reasonably justified. Even though the shapes of the PDFs are slightly skewed, the error correlations, *R*_s_, are reasonably high (*R* > 0.6) because the PDF shapes of $\Delta v_{\theta }^{C}$ and $\frac{A}{\sqrt 6 }$ have similar distributions. Since the sources of the uncertainty (explained in Appendix A) depend on the through-plane velocity, the PDF shapes are considered to depend on flow fields.

Although with increasing the frame number as shown in Fig. 7, the correlation values increased, the fitted lines does not converge to the unity line. Also, the uncertainty of the VAE may be contributed by the bias error of the VAE,  although the VAE can be that it was established under the condition that the velocity error is statistically random and spatially uniform with zero bias error as described in Eq. (13). The detail is explained in Appendix A. In all cases in Figs. 6 and 7, the fitted lines are all above the unity line, meaning that the VAE can conservatively alert the users for the uncertain measurements to avoid misdiagnosis.

## Conclusions

A novel method to estimate accuracy of VFM, named a posteriori “VFM accuracy estimation” (VAE), was proposed. By taking into account the discrepancy in velocities obtained by VFM and tissue-tracking measurements, VAE makes it possible to estimate uncertainty in each VFM measurement. VAE was validated by PIV using an in-house LV phantom. The uncertainty estimated by VAE agreed well with the uncertainty measured by PIV.

The clinical validity of VFM is expected to be improved by letting operators know the index of deviation from actual flows and by guiding them to better fields of view with this uncertain method of estimation.

## Figures and Tables

**Fig. 8:**
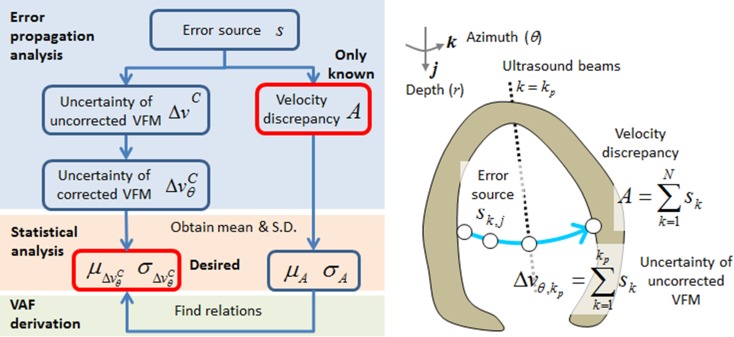
Schematic of VFM calculation domain in the LV, where velocity-error source, *s*, is distributed.

**Fig. 9:**
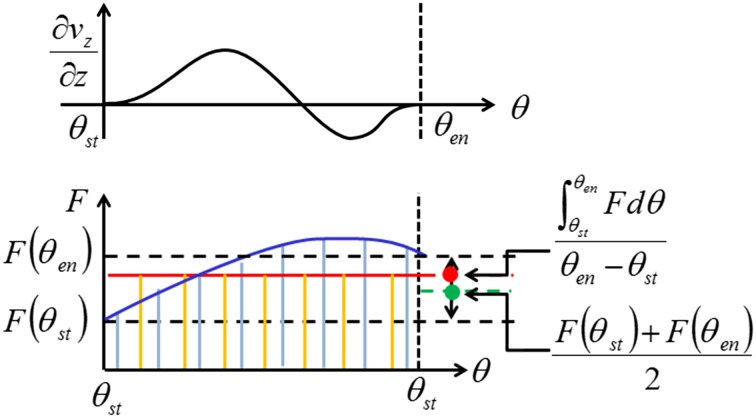
Schematics of the mean VFM-velocity error

**Fig. 10:**
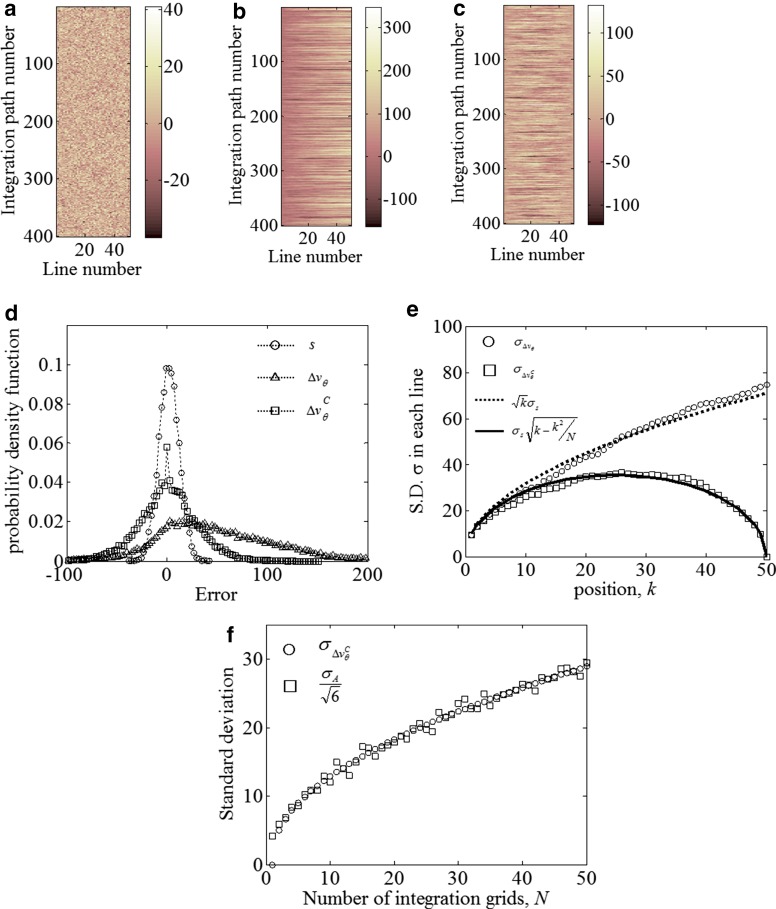
Simulation results. Contour plots of **a** vector error source, *s*, for S.D. of 10, **b** integrated vector-error source, and **c** corrected VFM error with bias errors. **d** Probability density function of uncorrected VFM error and corrected VFM uncertainty. **e** Standard deviations in each beam line. The S.D. was calculated using the samples in the same beam in the depth direction, **f** Standard deviations for the corrected VFM error and the estimated value as a function of *N*

## Appendix A: Formulation of proposed method

To derive Eq. (12), or to find unknowns $\mu_{{\Delta v_{\theta }^{C} }}$ and $\sigma_{{\Delta v_{\theta }^{C} }}$ using a measurable quantity, *A*, three steps are taken, as shown in Fig. 8. First, an error-propagation analysis is conducted to formulate the errors of uncorrected and corrected VFM velocities and the velocity discrepancy. Second, statistical analysis is conducted to obtain statistical quantities of each error. Third, the VAE uncertainty described in Eq. (12) was derived by finding the relation between the statistical quantities of the known velocity discrepancy, *A*, and the unknown corrected VFM error, $\Delta v_{\theta }^{C}$.

### Error-propagation analysis

The relationship between *A* and $\Delta v_{\theta }^{C}$ is formulated by considering the error-propagation model described in Eq. (17) on the basis of Eq. (2). The VFM calculation domain is schematically shown in Fig. 8, where velocity-error source, *s*, is distributed as follows,

17 $$v_{\theta }^{\text{st}} (r,\theta ) = v_{{\theta ,{\text{st}}}}^{T} (r) + \int_{{\theta_{\text{st}} }}^{\theta } {(\partial_{\theta } v_{\theta } + s)} {\text{d}}\theta .$$

The error source, *s*, includes all kind of errors, such as a CFM-measurement error and errors due to through-plane flows. By subtracting Eq. (2) from Eq. (17), the error terms can be derived as

18 $$\Delta v_{\theta } (r,\theta ) = \Delta v_{{\theta ,{\text{st}}}}^{T} (r) + \int_{{\theta_{\text{st}} }}^{\theta } s {\text{d}}\theta .$$

In the discretized form, the uncorrected VFM-velocity error at the *k*th position, $\Delta v_{\theta ,k}$, can be described as

19 $$\Delta v_{\theta ,k} = \Delta v_{{\theta ,{\text{st}}}}^{T} + \sum\limits_{i = 1}^{k} {s_{i} } ,$$

where the errors in uncorrected VFM measurement, $\Delta v_{\theta }$, and tissue tracking (TT) measurement, $\Delta v_{\theta }^{T}$, are defined as

20 $$\begin{aligned} \Delta v_{\theta } = v_{\theta }^{\text{st}} - v_{\theta } \hfill \\ \Delta v_{\theta }^{T} = v_{\theta }^{T} - v_{\theta } . \hfill \\ \end{aligned}$$

Similarly, the velocity discrepancy, *A*, is given by the accumulation of *s* as

21 $$A = \Delta v_{{\theta ,{\text{st}}}}^{T} - \Delta v_{{\theta ,{\text{en}}}}^{T} + \sum\limits_{i = 1}^{N} {s_{i} } .$$

Under the assumption that tissue-tracking uncertainty is much smaller than that of VFM error, i.e.,$\Delta v_{\theta }^{T} < \Delta v_{\theta }$, *A* can also be simplified and interpreted as the sum of VFM error source, *s*, along the integration path as

22 $$\Delta v_{\theta ,k} = \sum\limits_{i = 1}^{k} {s_{i} } ,$$

23 $$A = \sum\limits_{i = 1}^{N} {s_{i} } .$$

### Derivation of statistical properties

The descriptions of the mean and S.D. of $\Delta v_{\theta }$ and *A* are derived by considering the error-accumulation process described by Eqs. (22) and (23). In this derivation, it is assumed that the VFM-error source has a Gaussian distribution with a mean value of $\mu_{s}$ and a S.D. of $\sigma_{s}$. To consider the error accumulation, the simplest case for the mean value of *N* velocity-error sources, defined in Eq. (24), is first considered as

24 $$\overline{s} = \frac{1}{N}\sum\limits_{i = 1}^{N} {s_{i} } .$$

Under the Gaussian distribution assumption, the sampled mean, $\overline{s}$, also follows a Gaussian distribution with S.D. of $\sigma_{{\overline{s} }}$ on the basis of the central-limit theorem (Bendat et al. 2000):

25 $$\mu_{{\overline{s} }} \cong \mu_{s} ,$$

26 $$\sigma_{{\overline{s} }} = \frac{{\sigma_{s} }}{\sqrt N }.$$

The statistical properties of *A* are calculated, where *A* can be sampled from different integration paths with different depths, *r*. Similar to $\overline{s}$, the summed value, *A*, also has a Gaussian distribution with standard deviations of $\sigma_{A}$, which is simply given by multiplying $\sigma_{{\overline{s} }}$ by *N* as follows:

27 $$\mu_{A} = N\mu_{s} ,$$

28 $$\sigma_{A} = \sqrt N \sigma_{s} .$$

The mean and standard deviation of the corrected velocity error, $\Delta v_{\theta }$, ($\mu_{{\Delta v_{\theta }^{C} }}$ and $\sigma_{{\Delta v_{\theta }^{C} }}$) are derived as follows. From Eqs. (9) and (22), the error of the corrected VFM velocity, $\Delta v_{\theta }^{C}$, is expressed as

29 $$\begin{aligned} \Delta v_{\theta ,k}^{C} & = \sum\limits_{i = 1}^{k} {s_{i} } - \frac{k}{N}\sum\limits_{i = 1}^{N} {s_{i} } \\ & = \frac{N - k}{N}\sum\limits_{i = 1}^{k} {s_{i} } - \frac{k}{N}\sum\limits_{i = k + 1}^{N} {s_{i} } . \\ \end{aligned}$$

Therefore, the mean of $\Delta v_{\theta ,k}^{C}$ at the *k*th point in the *r* direction is given as

30 $$\begin{aligned} \left\langle {\Delta v_{\theta ,k}^{C} } \right\rangle_{r} & = \left\langle {\frac{N - k}{N}\sum\limits_{i = 1}^{k} {s_{i} } - \frac{k}{N}\sum\limits_{i = k + 1}^{N} {s_{i} } } \right\rangle_{r} \\ & = \frac{N - k}{N}k\left\langle s \right\rangle_{r} - \frac{k}{N}(N - k)\left\langle s \right\rangle_{r} \cong 0, \\ \end{aligned}$$

where the bracket, $\left\langle {\left( \cdot \right)} \right\rangle_{r}$, is an operator used for taking a mean of an arbitral quantity, $\left( \cdot \right)$, in the *r* direction (Fig. 8).

31 $$\left\langle {\left( \cdot \right)} \right\rangle_{r} = \frac{1}{M}\sum\limits_{j = 1}^{M} {\left( \cdot \right)_{j} } .$$

The number of samples, *M*, in direction *r* is assumed to be large enough to converge statistically. Therefore, according to Eq. (30), the mean of $\Delta v_{\theta ,k}^{C}$ in the entire domain is also zero. The additional subscript, *j*, denotes the *j*th integration path in the depth direction. The mean of $\Delta v_{\theta }^{C}$ is given as

32 $$\mu_{{\Delta v_{\theta }^{C} }} = \frac{1}{N}\sum\limits_{k = 1}^{N} {\left\langle {\Delta v_{\theta ,k}^{C} } \right\rangle_{r} } \cong 0.$$

S.D. of $\Delta v_{\theta }^{C}$ is defined as

33 $$\begin{aligned} \sigma_{{\Delta v_{\theta }^{C} }} & = \sqrt {\frac{1}{MN}\sum\limits_{k = 1}^{N} {\sum\limits_{j = 1}^{M} {\left( {\Delta v_{\theta ,k,j}^{C} } \right)^{2} } } } \\ & = \sqrt {\frac{1}{N}\sum\limits_{k = 1}^{N}  {\sigma_{{\Delta v_{\theta ,k}^{C} }}^{2} } }\\ \end{aligned}$$

where the S.D. of the *k*th integral point, $\Delta v_{\theta ,k}^{C}$, can be written as

34 $${\sigma_{{\Delta v_{\theta ,k}^{C} }}^{2} }  = \frac{1}{M}\sum\limits_{j = 1}^{M} {\left( {\Delta v_{\theta ,k,j}^{C} } \right)^{2} } .$$

From Eq. (29), the error of the corrected VFM velocity, $\Delta v_{\theta ,k}^{C}$, at the *k*th point consists of the following two independent variables.

$$\begin{aligned} p_{k} & = \frac{N - k}{N}\sum\limits_{i = 1}^{k} {s_{i} } \\ q_{k} & = \frac{k}{N}\sum\limits_{i = k + 1}^{N} {s_{i} } \\ \end{aligned}$$

where $p_{k}$ and $q_{k}$ also have Gaussian distributions with S.D. of $\sigma_{{p_{k} }}$ and $\sigma_{{q_{k} }}$, respectively, on the basis of the central-limit theorem (Bendat et al. 2000). Similar to that given by Eq. (26), the standard deviation of the summed values of *s* in each region can be written as

35 $$\sigma_{{p_{k} }} = \frac{N - k}{N}\sqrt k \sigma_{s} ,$$

36 $$\sigma_{{q_{k} }} = \frac{k}{N}\sqrt {N - k} \sigma_{s} .$$

Since $\Delta v_{\theta ,k}^{C}$ is the summation of $p_{k}$ and $q_{k}$, $\Delta v_{\theta ,k}^{C}$ also has a Gaussian distribution with $\sigma_{{\Delta v_{\theta ,k}^{C} }}$ given as

37 $$\begin{aligned} \sigma_{{\Delta v_{\theta ,k}^{C} }} & = \sqrt {\sigma_{{p_{k} }}^{2} + \sigma_{{q_{k} }}^{2} } \\ & = \sigma_{s} \sqrt {k - \frac{{k^{2} }}{N}} . \\ \end{aligned}$$

By substituting Eq. (37) into Eq. (33), the S.D. of $\Delta v_{\theta }^{C}$ becomes

38 $$\sigma_{{\Delta v_{\theta }^{C} }} = \sigma_{s} \sqrt {\frac{1}{N}\sum\limits_{k = 1}^{N} {\left( {k - \frac{{k^{2} }}{N}} \right)} } .$$

Using a series of constants (Spiegel 1999) as Eqs. (39) and (40) makes it possible to simplify Eq. (38) as Eq. (41) as follows:

39 $$\sum\limits_{k = 1}^{N} {k = \frac{N(N + 1)}{2}} ,$$

40 $$\sum\limits_{k = 1}^{N} {k^{2} = \frac{N(N + 1)(2N + 1)}{6}} ,$$

41 $$\sigma_{{\Delta v_{\theta }^{C} }} = \sigma_{s} \sqrt {\frac{{N^{2} - 1}}{6N}} .$$

Since *N* is usually more than 10, $N^{2}$ is much larger than one ($N^{2}$ ≫ 1). Therefore, the S.D. of $\Delta v_{\theta }^{C}$ can be further simplified as

42 $$\sigma_{{\Delta v_{\theta }^{C} }} = \sigma_{s} \sqrt {\frac{N}{6}} .$$

### Relationship between *A* and $\Delta v_{\theta }^{C}$

According to the error-propagation analysis, $\mu_{{\Delta v_{\theta }^{C} }}$ and $\sigma_{{\Delta v_{\theta }^{C} }}$ can be written as follows on the basis of properties of *A* derived from Eqs. (28), (32), and (42):

43 $$\mu_{{\Delta v_{\theta }^{C} }} \cong 0,$$

44 $$\sigma_{{\Delta v_{\theta }^{C} }} = \frac{{\sigma_{A} }}{\sqrt 6 }.$$

### Integration path selection for an LV-shaped domain

The proposed method has been constructed under conditions in which the number of integration points, *N*, is a constant as in the case of a simple rectangular domain. This condition needs to be satisfied even for LV-shaped domains. Accordingly, integration paths that have almost the same *N* were selected by following three steps schematically. First, a histogram of *N* in a frame is obtained. Second, the maximum value, *N*_max_, is found as

45 $$N_{\hbox{max} } - 2 \le N \le N_{\hbox{max} } + 2.$$

Third, the integration paths, which have *N* satisfying Eq. (45), are selected. Velocity discrepancy, *A*, values along the selected integration paths are used for the uncertainty estimation.

### Bias error of VAE

The bias error of the VAE is investigated in terms of error propagation due to the pure three-dimensionalities. The VAE is established under the condition that the velocity error is statistically random and spatially uniform with zero bias error (Eq. (13)). Given that the through-plane velocity is known with no measurement errors. The VFM velocity obtained by left, or right, integration can be described as

46 $$\begin{aligned} v_{\theta }^{\text{st(ideal)}} = v_{\theta ,st}^{T} + \int_{{\theta_{st} }}^{{\theta_{{}} }} {\left( { - r\frac{{\partial v_{r} }}{\partial r} - v_{r} - \frac{{\partial v_{z} }}{\partial z}} \right)} {\text{d}}\theta \hfill \\ v_{\theta }^{\text{en(ideal)}} = v_{\theta ,en}^{T} - \int_{\theta }^{{\theta_{st} }} {\left( { - r\frac{{\partial v_{r} }}{\partial r} - v_{r} - \frac{{\partial v_{z} }}{\partial z}} \right)} {\text{d}}\theta . \hfill \\ \end{aligned}$$

The superscript (ideal) denotes this special case. Both velocities and corrected velocities must be identical and they have a true value.

47 $$v_{\theta }^{\text{st(ideal)}} = v_{\theta }^{\text{en(ideal)}} = v_{\theta }^{\text{C(ideal)}} = v_{\theta } .$$

Therefore, the difference between $v_{\theta }^{\text{st(ideal)}}$ and $v_{\theta }^{\text{en(ideal)}}$ is zero:

48 $$\begin{aligned} & v_{\theta }^{\text{st(ideal)}} - v_{\theta }^{\text{en(ideal)}} \\ & \quad = v_{{\theta ,{\text{st}}}}^{T} - v_{{\theta ,{\text{en}}}}^{T} + \int_{{\theta_{\text{st}} }}^{{\theta_{\text{en}} }} {\left( { - r\frac{{\partial v_{r} }}{\partial r} - v_{r} - \frac{{\partial v_{z} }}{\partial z}} \right)} {\text{d}}\theta \\ & \quad = 0. \\ \end{aligned}$$

When through-plane velocity is unknown, as for a real situation, the difference between $v_{\theta }^{\text{st}}$ and $v_{\theta }^{\text{en}}$ is described as

49 $$\begin{aligned} v_{\theta }^{\text{st}} - v_{\theta }^{\text{en}} & = A \\ & = v_{{\theta ,{\text{st}}}}^{T} - v_{{\theta ,{\text{en}}}}^{T} + \int_{{\theta_{\text{st}} }}^{{\theta_{\text{en}} }} {\left( { - r\frac{{\partial v_{r} }}{\partial r} - v_{r} } \right)} {\text{d}}\theta . \\ \end{aligned}$$

From Eqs. (48) and (49), velocity discrepancy *A* can be written as

50 $$A = \int_{{\theta_{\text{st}} }}^{{\theta_{\text{en}} }} {\left( {\frac{{\partial v_{z} }}{\partial z}} \right)} {\text{d}}\theta .$$

The corrected velocity, $v_{\theta }^{C}$, is also calculated from Eq. (9) as

51 $$v_{\theta }^{C} = v_{{\theta_{\text{st}} }}^{T} + \int_{{\theta_{\text{st}} }}^{{\theta_{{}} }} {\left( { - r\frac{{\partial v_{r} }}{\partial r} - v_{r} } \right)} {\text{d}}\theta - \frac{{\theta - \theta_{\text{st}} }}{{\theta_{\text{en}} - \theta_{\text{st}} }}A.$$

The error of the corrected VFM, $\Delta v_{\theta }^{C}$, is described on the bases of Eqs. (20), (46), (47), (50), and (51) as

52 $$\begin{aligned} \Delta v_{\theta }^{C} & = v_{\theta }^{C} - v_{\theta }^{{}} \\ & = \int_{{\theta_{\text{st}} }}^{{\theta_{{}} }} {\left( {\frac{{\partial v_{z} }}{\partial z}} \right)} {\text{d}}\theta - \frac{{\theta - \theta_{\text{st}} }}{{\theta_{\text{en}} - \theta_{\text{st}} }}\int_{{\theta_{\text{st}} }}^{{\theta_{\text{en}} }} {\left( {\frac{{\partial v_{z} }}{\partial z}} \right)} {\text{d}}\theta . \\ \end{aligned}$$

The mean of the velocity error along an integration path, $\left\langle {\Delta v_{\theta }^{C} } \right\rangle_{\theta }$, is then derived as in Eq. (53), since it is not easy to formulate the mean value of the VFM error for an entire domain,

53 $$\left\langle {\Delta v_{\theta }^{C} } \right\rangle_{\theta } = \frac{1}{{r(\theta_{\text{en}} - \theta_{\text{st}} )}}\int_{{\theta_{\text{st}} }}^{{\theta_{\text{en}} }} {\Delta v_{\theta }^{C} r} {\text{d}}\theta = \frac{{\int_{{\theta_{\text{st}} }}^{{\theta_{\text{en}} }} F {\text{d}}\theta }}{{\theta_{\text{en}} - \theta_{\text{st}} }} - \frac{{F(\theta_{\text{en}} ) - F(\theta_{\text{st}} )}}{2},$$

where, the function, *F*, is defined as:

54 $$F(\theta ) = \int {\frac{{\partial v_{z} }}{\partial z} \, {\text{d}}\theta}. $$

Under the no-slip condition, the first velocity derivatives, ${{\partial v_{z} } \mathord{\left/ {\vphantom {{\partial v_{z} } {\partial z}}} \right. \kern-0pt} {\partial z}}$, were set to zero at the wall. Figure 9 shows a schematic of the behavior of the mean value in Eq. (54). The mean of the corrected VFM error can be described as the difference between the average of $F(\theta_{\text{en}} )$ and $F(\theta_{\text{st}} )$ and the mean of $F\left( \theta \right)$. Strictly speaking, they are different, but they are close. The difference contributes to the uncertainty of the VAE. The validity of the VAE is also demonstrated in the next section.

### Numerical verification for simple rectangular domain

The formulation for the estimation of VFM uncertainty in the previous section was validated using a Monte Carlo simulation in MATLAB. Only error-source components were considered to be examined by the error-propagation analysis using Eq. (22). The simulation domain was set as rectangular. The error source, *s*, is simply set as a normally distributed random number with S.D. of $\sigma_{s}$. The simulation conditions are listed in Table 1.

**Table 1:** Simulation conditions

Properties	Symbols	Values
Number of integration points	*N*	50
Number of integration paths	*M*	400
Mean of *s*	*µ*_*s*_	2
SD of *s*	σ_*s*_	10

Figure 10a shows randomly distributed *s* values for $\sigma_{s}$ of 10 with bias error, $\mu_{s}$, of 2. The distribution of the uncorrected velocity error, $\Delta v_{\theta }^{{}}$, was calculated by integrating error sources using Eq. (22), as shown in Fig. 10b. Due to the accumulation process, a stripe-formed error can be found in Fig. 10b. The stripes in the azimuth direction are formed along the integration paths, because $\Delta v_{\theta ,k}^{{}}$ in the azimuthal direction becomes correlated by the integration. Near the end wall (*k* = *N*), this trend is stronger, and the uncertainty level is $\sqrt N$ times the original uncertainty level, as given by Eq. (28). The accumulated error is monotonically increased due to the bias error.

The distribution of the uncorrected velocity error, $\Delta v_{\theta }^{C}$, was calculated from Eq. (29) and is plotted in Fig. 10c. By the correction, the stripe-formed error is suppressed in entire regions, especially near the end wall. Moreover, the accumulated bias error was eliminated in accordance with Eq. (32).

The PDFs of VFM error source, *s*, uncorrected VFM error, $\Delta v_{\theta }^{{}}$, and corrected VFM error, $\Delta v_{\theta }^{C}$, in the entire domain were calculated and are plotted in Fig. 10d. The PDF of $\Delta v_{\theta }^{{}}$ stretched out due to the accumulated bias errors. However, the PDF of $\Delta v_{\theta }^{C}$ becomes narrower, indicating that the correction method effectively reduced the bias errors. The PDF of $\Delta v_{\theta }^{{}}$ becomes wider than that of *s* because of the striped-form error. As illustrated in Fig. 10e, the accumulated error strongly depends on the integration position, *k*, because the error accumulates. This trend agrees with the theoretical estimation based on the central-limit theorem (Eq. (28)) given as

55 $$\sigma_{{\Delta v_{\theta ,k}^{{}} }} = \sigma_{s} \sqrt k$$

Since the error-correction method reduces the error in accordance with the distance from the wall, the magnitude of the error becomes large around the center of the domain.

The uncertainty-estimation method described in Eq. (44) was validated as a function of *N*, since the error-accumulation effect depends on the number of integrations, *N*. Figure 10f shows the S.D. of the corrected VFM error, $\sigma_{{\Delta v_{\theta }^{C} }}$, and the estimated value ($= \sigma_{A} /\sqrt 6$) as a function of *N*. Here, $\sigma_{{\Delta v_{\theta }^{C} }}$ was calculated using corrected VFM error samples in the entire two-dimensional domain, and the S.D. of the velocity discrepancy, $\sigma_{A}$, was calculated using the samples in the one-dimensional depth direction at the end wall. As the number of integration points, *N*, increases, the S.D. of the corrected VFM error also increases due to the region far from the walls. Moreover, the estimated uncertainty agrees well with the resultant error, $\sigma_{{\Delta v_{\theta }^{C} }}$. The uncertainty-estimation scheme for the corrected VFM velocity was validated for the simple rectangular case.

## Appendix B

The resultant VFM velocity calculated by the ultrasound equipment, $\overrightarrow {{v^{\text{eqp}} }}$, was modified by simply multiplying the measured vectors by a correction factor, *C*_*f*_, given by Eq. (16). For the beam direction, the ultrasound equipment detects the Doppler-shift frequency, $\Delta f$, and calculated velocity using the carrier frequency, $f{}_{0}$, as

56 $$v_{r}^{\text{eqp}} = \frac{\Delta f}{{f_{0} }}\frac{{c_{\text{b}} }}{2},$$

although real velocity, $v_{r}^{{}}$, is calculated as

57 $$v_{r}^{{}} = \frac{\Delta f}{{f_{0} }}\frac{{c_{\text{P}} }}{2}.$$

The relationship between $v_{r}^{\text{eqp}}$ and $v_{r}^{{}}$ is thus simply derived as

58 $$v_{r}^{{}} = v_{r}^{\text{eqp}} C_{\text{f}} .$$

For the azimuthal direction, VFM velocity, $v_{\theta }^{\text{eqp}}$, is calculated by the ultrasound equipment on the basis of the following equation:

59 $$v_{\theta }^{\text{eap}} (r,\theta ) = v_{{\theta ,{\text{st}}}}^{\text{eap}} (r) + \int_{{\theta_{\text{st}} }}^{\theta } {\partial_{\theta } v_{\theta }^{\text{eap}} } {\text{d}}\theta .$$

Each term in the equation is examined to derive the correction for the speed of sound. The first term represents wall velocity, $v_{{\theta ,{\text{st}}}}^{\text{eap}}$, which is calculated by tissue-speckle tracking using B-mode images. This is because the scale of entire B-mode images is simply reduced by $C_{\text{f}}$ because the set speed of sound is lower than the real speed or that in PEG400.

60 $$r = r^{\text{eqp}} C_{\text{f}}$$

Thus, the corrected wall velocity should be

61 $$v_{{\theta ,{\text{st}}}}^{{}} = v_{{\theta , {\text{st}}}}^{\text{eap}} C_{\text{f}} .$$

For the second term, the inside of the integration part in Eq. (59) is discretized and simplified using Eqs. (58) and (60) as

62 $$\begin{aligned} \partial_{\theta } v_{{\theta ,{\text{st}}}}^{\text{eqp}} & \cong - r^{\text{eqp}} \frac{{\Delta v_{r}^{\text{eqp}} }}{{\Delta r_{{}}^{\text{eqp}} }} - v_{r}^{\text{eqp}} \\ & \cong \frac{{\partial_{\theta } v_{\theta }^{{}} }}{{C_{\text{f}} }}. \\ \end{aligned}$$

By substituting Eqs. (61) and (62) into Eq. (59), the correction can be written as

63 $$v_{\theta}^{{}} = v_{\theta}^{\text{eqp}} C_{\text{f}}$$

The correction using Eq. (16) is thus justified by Eqs. (58) and (63).
